# A signature of 24 aging‑related gene pairs predict overall survival in gastric cancer

**DOI:** 10.1186/s12938-021-00871-x

**Published:** 2021-04-06

**Authors:** Yankai Zhang, Yichao Yan, Ning Ning, Zhanlong Shen, Yingjiang Ye

**Affiliations:** 1 Department of Gastroenterological Surgery, Laboratory of Surgical Oncology, Beijing Key Laboratory of Colorectal Cancer Diagnosis and Treatment Peking University People’s Hospital, No.11 Xizhimen South Street, Xicheng, Beijing, 100044 People’s Republic of China; 2 Department of Gastrointestinal Surgery, Peking University International Hospital, No.1 Life Park Road, Life Science Park of Zhong Guancun, Changping, Beijing, 102206 People’s Republic of China

**Keywords:** Gastric cancer, Aging-related gene pairs, Prognosis, Nomogram

## Abstract

**Background:**

Aging is the major risk factor for most human cancers. We aim to develop and validate a reliable aging-related gene pair signature (ARGPs) to predict the prognosis of gastric cancer (GC) patients.

**Methods:**

The mRNA expression data and clinical information were obtained from two public databases, The Cancer Genome Atlas (TCGA) dataset, and Gene Expression Omnibus (GEO) dataset, respectively. The best prognostic signature was established using Cox regression analysis (univariate and least absolute shrinkage and selection operator). The optimal cut-off value to distinguish between high- and low-risk patients was found by time-dependent receiver operating characteristic (ROC). The prognostic ability of the ARGPS was evaluated by a log‐rank test and a Cox proportional hazards regression model.

**Results:**

The 24 ARGPs were constructed for GC prognosis. Using the optimal cut-off value − 0.270, all patients were stratified into high risk and low risk. In both TCGA and GEO cohorts, the results of Kaplan–Meier analysis showed that the high-risk group has a poor prognosis (*P* < 0.001, *P* = 0.002, respectively). Then, we conducted a subgroup analysis of age, gender, grade and stage, and reached the same conclusion. After adjusting for a variety of clinical and pathological factors, the results of multivariate COX regression analysis showed that the ARGPs is still an independent prognostic factor of OS (HR, 4.919; 95% CI 3.345–7.235; *P* < 0.001). In comparing with previous signature, the novel signature was superior, with an area under the receiver operating characteristic curve (AUC) value of 0.845 vs. 0.684 vs. 0.695. The results of immune infiltration analysis showed that the abundance of T cells follicular helper was significantly higher in the low-risk group, while the abundance of monocytes was the opposite. Finally, we identified and incorporated independent prognostic factors and developed a superior nomogram to predict the prognosis of GC patients.

**Conclusion:**

Our study has developed a robust prognostic signature that can accurately predict the prognostic outcome of GC patients.

**Supplementary Information:**

The online version contains supplementary material available at 10.1186/s12938-021-00871-x.

## Background

Globally, gastric cancer (GC) is an important public health problem, and its morbidity and mortality are at the forefront of cancer [1, 2]. As research continues, treatment approaches for GC have been refined and optimized. However, due to the occult course of GC, most patients who are first diagnosed are already at an advanced stage, with a 5-year survival rate of only about 30% [3]. In addition, GC is a highly heterogeneous cancer, has multiple histological types and each type has a unique biological behavior [4, 5]. It is well known that the most common prognostic tool used by clinicians is the American Joint Committee on Cancer (AJCC) tumor node metastasis (TNM) stage system [6]. However, some patients with GC have the same TNM stage and finally get different clinical outcomes, which show that the AJCC stage system ignores the biological heterogeneity of the tumor. Therefore, novel and powerful prognostic signature is needed in clinical work to accurately identify high-risk patients in order to improve survival outcomes in GC patients.

Aging is the process of loss and degeneration of the body from constitutive substances and tissue structures to physiological functions [7, 8]. Previous studies have shown that aging is the risk factor for many diseases, such as heart disease [9], neurodegenerative diseases [10], type 1 diabetes [11] and cancer [12]. Interestingly, aging-related genes (AGs) have two different biological roles in regulating cell senescence. It can inhibit tumor growth by regulating the senescence of tumor cells, and can also promote tumor invasion and metastasis [13–16]. Selecting those gene sets with potential biological significance for the development of predictive models can provide new insights for individualized treatment. Given the key role of aging in the development of GC, we selected a set of AGs to build a gene signature to distinguish different risk groups, which are of great benefit in selecting personalized treatment plans for patients.

The rapid development of high-throughput sequencing technologies and bioinformatics tools has allowed us to study cancer in greater depth. For GC, many previous studies have established gene signature to predict the prognosis of patients [17–19]. However, for various reasons (technical limitations and diversity of data types) these gene signature have not been widely used in daily clinical work. Fortunately, in order to overcome above problems and make the developed gene signatures available to clinicians as early as possible, researchers have developed a new algorithm based on the relative ranking of gene expression levels, and many studies using this algorithm have yielded reliable results [20–22].

Therefore, the main focus of this study is to construct a robust and stable individualized prognostic signature based on AGs using a new algorithm. In addition, the robustness of our signature was validated using another independent cohort. Subsequently, we also compared the performance of the new signature with previously published prognostic models for GC.

## Results

### Construction of ARGPs signature

**Fig. 1:**
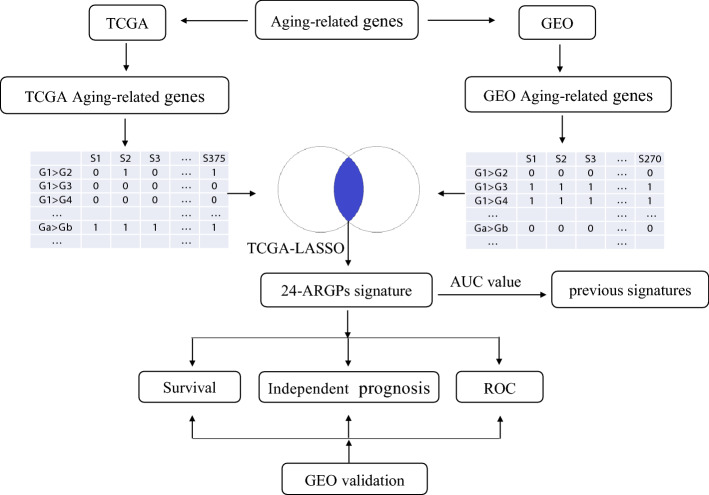
Flowchart of the research procedure in this study

The analysis process of present study is shown in Fig. 1. As described in the method, a total of 155 ARGPs related to prognosis was identified. Next, we applied a Lasso‐penalized Cox regression to further reduce the number of ARGPs in the prognostic model. Finally, we filtered to obtain 24 ARGPs and their corresponding coefficients (Table 1). Based on the score of each gene pair and the corresponding coefficient, the following formula was constructed to calculate the risk score:

**Table 1:** Prognostic signature consists of 24 aging-related gene pairs

Signature	Gene A	Gene B	Coefficient
Pair1	TP53	PDGFRB	− 0.097
Pair2	E2F1	PTGS2	− 0.090
Pair3	E2F1	SERPINE1	− 0.043
Pair4	STAT5B	FOXO1	0.256
Pair5	NGFR	ADCY5	0.067
Pair6	PDGFB	TFAP2A	0.069
Pair7	SST	BCL2	− 0.462
Pair8	PRKCD	FEN1	0.219
Pair9	HIF1A	MIF	0.059
Pair10	NR3C1	PIK3R1	0.221
Pair11	BRCA2	RGN	− 0.042
Pair12	CEBPA	FEN1	0.001
Pair13	FAS	IL6	− 0.174
Pair14	BAX	SERPINE1	− 0.388
Pair15	FOXO1	CDKN2B	− 0.370
Pair16	MSRA	PDGFRA	− 0.142
Pair17	RECQL4	NUDT1	− 0.072
Pair18	UCHL1	CDKN2B	0.188
Pair19	SNCG	PPARGC1A	0.120
Pair20	GSR	CDKN1A	− 0.046
Pair21	PIK3R1	EFEMP1	− 0.123
Pair22	PIK3R1	PDGFRA	− 0.075
Pair23	MXD1	PYCR1	0.073
Pair24	DBN1	SERPINE1	− 0.217

Risk score = (− 0.097 * TP53_PDGFRB) + (− 0.090 * E2F1_PTGS2) + (− 0.043 * E2F1_SERPINE1) + (0.256 * STAT5B_FOXO1) + (0.067 * NGFR_ADCY5) + (0.069 * PDGFB_TFAP2A) + (− 0.462 * SST_BCL2) + (0.219 * PRKCD_FEN1) + (0.059 * HIF1A_MIF) + (0.221 * NR3C1_PIK3R1) + (− 0.042 * BRCA2_RGN) + (0.001 * CEBPA_FEN1) + (− 0.174 * FAS_IL6) + (− 0.388 * BAX_SERPINE1) + (− 0.370 * FOXO1_CDKN2B) + (− 0.142 * MSRA_PDGFRA) + (− 0.072 * RECQL4_NUDT1) + (0.188 * UCHL1_CDKN2B) + (0.120 * SNCG_PPARGC1A) + (− 0.046 * GSR_CDKN1A) + (− 0.123 * PIK3R1_EFEMP1) + (− 0.075 * PIK3R1_PDGFRA) + (0.073 * MXD1_PYCR1) + (− 0.217 * DBN1_SERPINE1).

### Validation and assessment of the established ARGPs signature

**Fig. 2:**
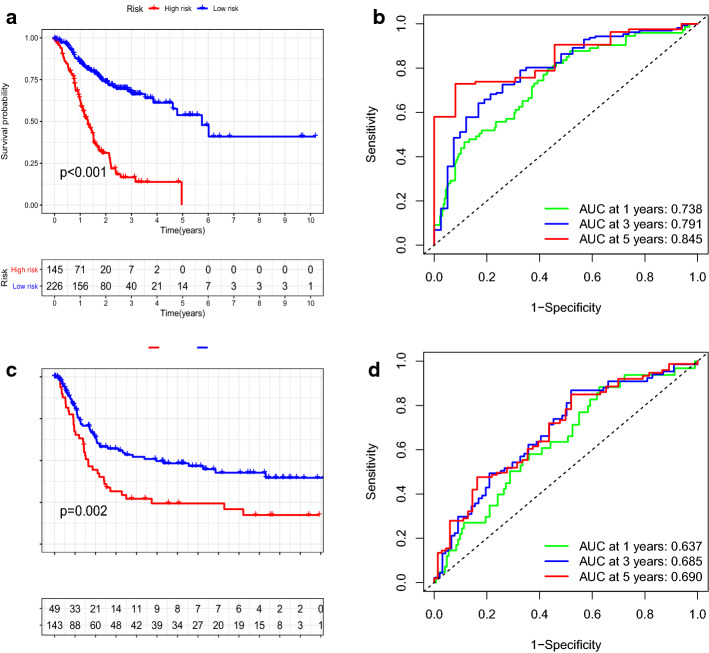
Construction and validation of aging-related gene pair signatures (ARGPs). **a** Survival curves of high- and low-risk groups separated by ARGPs in the TCGA cohort. **b** Receiver operating characteristic (ROC) curves for survival risk predicted by ARGPs for 1-, 3-, and 5-year follow-ups. **c** Survival curve of high- and low-risk groups separated by ARGPs in the GEO cohort. **d** ROC analysis for survival rate predicted by ARGPs for 1-, 3-, and 5-year follow-ups

According to the above formula, the risk score for all patients was calculated in the TCGA dataset. The optimal cut-off points for the risk score using ROC curve analysis were − 0.270, which can categorize the patients into two groups (high risk vs. low risk) (Additional file 1: Figure S1). The K–M curve and log-rank test showed that patients in the high-risk group have a shorter survival time than in the low-risk group (*P* < 0.001, Fig. 2a). Next, we used the same cut-off value to divide patients in the GEO dataset into two groups, and got the same results (*P* = 0.002, Fig. 2c). In addition, to further evaluate the prediction accuracy of ARGPs signature, a time-dependent ROC curve analysis was performed. In the TCGA cohort, the AUC values of the prognostic models predicted by 1-year, 3-year and 5-year survival rates were 0.738, 0.791 and 0.845, respectively (Fig. 2b). This demonstrated that the predictive performance of our established prognostic signature was reliable. The AUC values for OS in the GEO cohort at 1 year, 3 years and 5 years were 0.637, 0.685 and 0.690, respectively (Fig. 2d). These results were sufficient to confirm that the prognosis model can accurately predict the prognosis of GC patients.

**Fig. 3:**
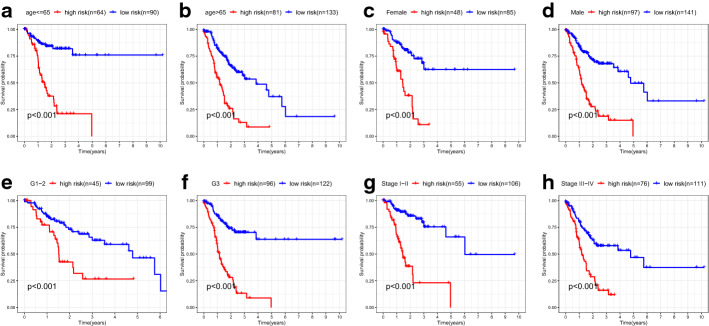
The Kaplan–Meier curves of patients with gastric cancer stratified by age (**a**, **b**), gender (**c**, **d**), grade (**e**, **f**), and stage (**g**, **h**)

Subsequently, we performed a stratified survival analysis to determine whether our model still had predictive value in different subgroups of clinicopathological parameters. As expected, the K–M curves illustrated that the signature was a stable prognostic marker for patients with GC stratified by age (≤ 65 or > 65), gender (female or male), grade (G1–2 or G3), and stage (I–II or III–IV) (Fig. 3).

**Fig. 4:**
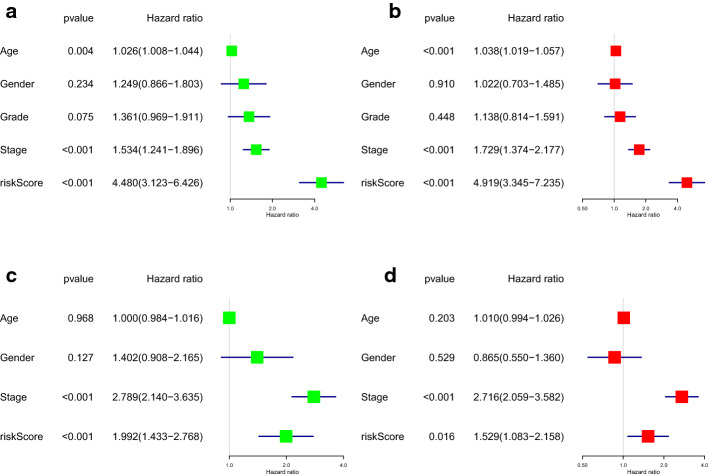
Univariate (**a**) and multivariate (**b**) analyses of prognostic factors in TCGA cohort and univariate (**c**) and multivariate (**d**) analyses of prognostic factors in GEO cohort identified the signature as an independent prognostic factor

In addition, univariate and multivariate Cox regression analyses were performed in the TCGA and GEO datasets to assess whether the prognostic power of ARGPs signature was independent of other clinicopathological parameters (age, sex, stage, grade). After two COX regression analyses, the risk score remained an independent prognostic indicator in the TCGA cohort (HR = 4.919, 95% CI [3.345–7.235], *P* < 0.001; Fig. 4a, b). As shown in Fig. 4c, d, the independent value of the ARGPs signature risk score had been verified in the GEO dataset (HR = 1.529, 95% CI [1.083–2.158], *P* = 0.016).

### Comparison of the performance of ARGPs signature and existing models in survival prediction

**Fig. 5:**
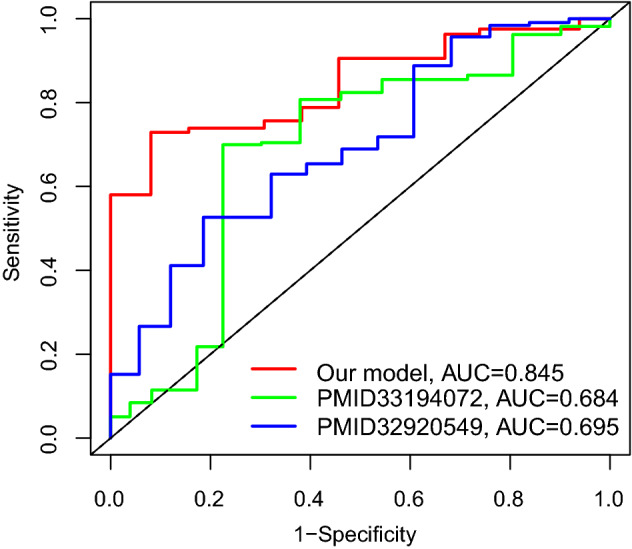
Comparison of ROC analysis between our model and two other existing models in predicting 5-year overall survival

We further compared the predictive performance of ARGPs signatures with two previously published mRNA signatures: the RNA-binding protein-related gene signature established by Zhou et al. [23] and the metabolic-related gene signature established by Wen et al. [24] using the same TCGA patient cohort. As shown in Fig. 5, the AUC at 5 years of OS for the ARGPs was 0.845, which was significantly higher than that of RNA-binding proteins signature (AUC = 0.684) and metabolic-related gene signature (AUC = 0.695). Collectively, the above result indicated that our model was a promising tool for predicting the prognosis of GC patients.

### Functional and pathway enrichment analysis

Additional file 2: Figure S2a shows the results of GO enrichment analysis. These genes were significantly enriched in response to oxidative stress, aging, apoptotic signaling pathway, regulation of reactive oxygen species metabolic process, negative regulation of cell proliferation, reproductive structure development. The KEGG pathway analysis revealed that these genes were highly associated with the pathways in cancer, HTLV-I infection, Kaposi sarcoma-associated herpesvirus infection, AGE–RAGE signaling pathway in diabetic complications, cellular senescence (Additional file 2: Figure S2b).

### Immune cell infiltration in different risk groups

**Fig. 6:**
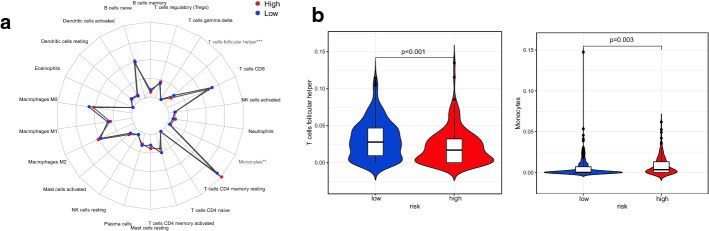
The relative fraction of infiltrated immune cells in different risk groups in the TCGA dataset. **a** Radar plot of the difference in the abundance of 22 immune cells in tumor tissue in the two risk groups. **b** The abundance distribution of T cells follicular helper and monocytes within the high-risk group and the low-risk group

Tumor-infiltrating immune cells have been shown to correlate with the prognosis of many cancer patients [25, 26]. We applied the CIBERSORT algorithm to estimate the relative abundance of 22 immune cells within different risk groups. The radar charts depict a comparative summary of various immune cells in these two risk groups (Fig. 6a). We found that immune cells, such as T cells follicular helper and monocytes were enriched in different risk groups. Among them, T cells follicular helper was significantly and highly expressed in the low-risk group, and the monocytes were highly expressed in the high-risk group (Fig. 6b).

### Establishment and evaluation of nomogram for predicting the prognosis of GC patients

**Fig. 7:**
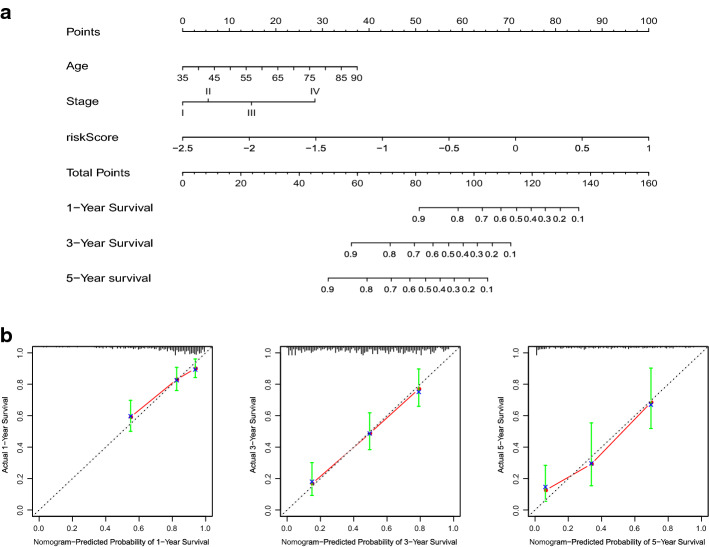
Construction and evaluation of nomogram. **a** A nomogram that integrated the age, stage and risk score. **b** The calibration curves for 1-, 3-, and 5-year overall survival

Multivariate COX regression analysis showed that in addition to the risk score, age and stage are also independent prognostic factors for OS. To further improve the accuracy of clinicians in predicting patient prognosis, we constructed a practical and novel nomogram tool combining all parameters with independent prognostic significance (Fig. 7a). The total score was obtained by calculating the patient's score for each parameter separately, and by making a vertical line downward from the total score on the nomogram, the clinician can easily derive the probability of OS at 1, 3 and 5 years. In addition, we also evaluated the performance of the nomogram. The calibration curves consisting of calibration points were very close to the standard curves (Fig. 7b). This was sufficient to show that our nomogram prediction performance was very strong, especially when predicting the 3-year OS.

## Discussion

In this study, the 24 ARGPs obtained from the screening were constructed into a new prognostic signature and its performance was further validated in an independent GEO cohort. The results showed the robust performance of our gene signature in predicting the prognosis of GC patients and accurately distinguishing high-risk populations. Then, subgroup survival analysis of age, sex, grade and stage also verified the stability of the signature. In addition, multivariate Cox analysis showed that the risk score was an independent prognostic factor for GC patients. In addition, the new prognostic signature identified performed well compared to the two published prognostic signatures, which is an important strength of this study. Finally, our nomogram constructed by combining risk score and clinicopathological parameters with independent prognostic significance can improve the predictive accuracy of OS in patients with GC.

Due to the heterogeneity of GC and the technical noise caused by cross-platform sequencing, the previously established prognostic signature needs to standardize the mRNA expression profile, which is a difficult but necessary step in data analysis. However, our study differs from previous literature in the building of prognostic signatures. The construction of the prognostic signature is based on gene pairs rather than single genes in this study. This method of data analysis using gene expression values for pairwise comparison is a novel algorithm that can overcome the above-mentioned problems. The use of novel algorithms to construct signatures is an advantage in this study, and has been proven to be reliable in previous studies [27]. ARGPs signature not only have a predictive prognostic function, but also reduce the detection process to some extent. For instance, both genes, RECQL4 and NUDT1, were overexpressed in GC tissues. Increased RECQL4 expression was associated with poor prognosis in GC patients [28]. The overexpression of NUDT1 was closely related to the increased depth of invasion and lower survival rate of GC patients [29]. These two mRNAs formed an ARGP in this study, and when the expression value of RECQL4 was lower than the expression level of NUDT1, a high-risk score was acquired, leading to poorer prognostic outcomes. The results require only a brief comparison of the magnitude of expression values for the constituent genes in the prognostic gene pairs, without the need for specific expression data. Thus, its feasibility is better than traditional gene signature detection. This novel signature is composed of 36 unique AGs, some of which have been intensively studied in GC and other cancers. For example, previous studies have shown that the expression of PDFGRB was related to lymphatic metastasis and poor prognosis of GC [30]. CDKN2B is highly expressed in GC tissues compared with adjacent tissues, and can be used as a prognostic marker for GC [31]. Ai et al.’s study showed that PIK3R1 overexpression can promote the progression of hepatocellular carcinoma [32]. The results of Irene Arroyo-Solera et al. [33] showed that high SERPINE1 expression promotes tumor invasion and metastasis, and is also associated with poor prognosis in head and neck squamous cell carcinoma patients. The above evidence is sufficient to suggest that ARGPs are not surprising as a prognostic signature for GC.

Recently, a lot of evidence supports the fact that the tumor microenvironment plays a huge role in determining the occurrence and development of tumors [34–36]. The results of Zeng et al. on the role of tumor immune infiltrating cells in GC suggest that it can be used as independent prognostic markers [37, 38]. Therefore, in this study we compared the differences in the abundance of tumor immune infiltrating cells between the high-risk and low-risk groups, and the results showed that two types of immune cells, T cells follicular helper, monocytes, differed between the two groups. There into, T cells follicular helper was more enriched in the low-risk group. Pan et al. found that T cells follicular helper was associated with poorer results in clear cell renal cell carcinoma [39]. The biological significance of T cells follicular helper in GC needs further study. In addition, compared with the low-risk group, the high-risk group had higher levels of monocytes. Increased monocytes have a negative impact on tumor immunity and are related to the poor prognosis of GC [40].

Nomogram is a powerful and easy-to-use tool that has been widely used in previous biomedical studies [41–43]. The clinicopathological parameters we included in the nomogram were all factors with independent prognostic significance obtained by multivariate Cox regression analysis; in this way, the nomogram plot is more convincing for predicting the prognosis of GC patients, and we also tested the accuracy of the tool using calibration plot curves. Although studies have been developed to predict prognosis nomogram plots for GC patients, this is the first one to be constructed in conjunction with ARGPs signature, providing new ideas for the development and use of nomograms.

Nevertheless, some limitations to our study should be acknowledged. First of all, we cannot avoid that our research results are based on retrospective analysis, and the results should be verified in prospective studies. Second, functional experimental studies should be conducted on these AGs to progressively elucidate the function of these ARGPs in GC.

## Conclusion

In summary, we have developed a robust ARGPs signature using a new algorithm. It can still accurately predict the prognosis of individual patients in independent GEO data set. In addition, its performance was better than previously published prognostic models.

## Materials and methods

### Data sources and AGs acquisition

The preprocessed level 3 RNA-seq data and associated clinical information of GC were mined from TCGA data portal. Similarly, independent validation dataset GSE15459 was downloaded from GEO, including gene expression files and clinical follow-up information of 192 GC sample. 307 human AGs were obtained from the human aging genome resource (HAGR, http://genomics.senescence.info/genes/). Probe IDs correctly changed to the corresponding gene symbols based on the annotated information on the platform without further standardization. If multiple probe IDs are mapped to a single gene symbol, the average value is calculated as the expression value of the gene for further analysis. Expression data for 307 AGs were extracted from the TCGA cohort and the GSE15459 dataset, respectively, and the AGs common to both datasets were thus obtained.

### Construction of a prognostic gene signature based on ARGPs

First, the median absolute deviation (MAD) < 0.5 was used as a filtering condition to discard those AGs that were imbalanced in distribution or had fairly little mutations [21]. The values of gene expression in a given sample or profile were compared pairwise to obtain a score for each ARGP. Briefly, the value was assigned as 1 when the first AG exhibit higher expression relative to the second AG in each ARGP; otherwise, the output was 0. In addition, we also filter out those ARGPs with a ratio of “0” or “1” < 20%. This novel algorithm was one of the strengths of this study because ARGP was derived from pairwise comparisons and was based entirely on gene expression in the same sample, which can overcome the bias associated with sequencing across different platforms and does not require additional processing of the data. A total of 1278 ARGPs were used as initial candidate factors for prognostic prediction in two datasets. Through univariate Cox regression and the Kaplan–Meier (K–M) method, a summary of 155 ARGPs related to the prognosis of survival were screened out (*P* < 0.05). To get a more practical model, we used least absolute shrinkage and selection operator (LASSO) regression to eliminate highly correlated ARGPs. Finally, 24 ARGPs were reserved for constructing prognostic signature. For every patient, the risk score was obtained according to the following model formula we constructed:

$${\text{Risk score}} = {\text{ coef}}\left( {{\text{gene}}1} \right) *{\text{Exp}}\left( {{\text{gene}}1} \right) \, + {\text{Coef }}\left( {{\text{gene}}2} \right)*{\text{ Exp }}\left( {{\text{gene}}2} \right) \, + \, \cdots \, + {\text{ coef}}\left( {{\text{genen}}} \right)*{\text{ Exp }}\left( {{\text{genen}}} \right),$$

where “coef” was the coefficient estimated from the LASSO regression analysis and “Exp” represents the score of the ARGP. We used the R package “survival” and “survivalROC” to access the best cut-off value of a risk score by the time-dependent ROC curve analysis. We then categorized the GC patients into high- and low-risk groups, according to the best cut-off value.

### Evaluation of the prognostic performance of the ARGPs signature

Survival curves for OS were generated according to the K–M method and the log-rank test was applied to compare the differences between low- and high-risk groups. The predictive ability of the model was then determined by the area under the ROC curve (AUC). Univariate and multivariate Cox analysis were performed to estimate the influence of risk score model on prognosis and other clinicopathological factors in the TCGA dataset. Hazard ratios (HRs) with their corresponding 95% confidence intervals (CIs) were obtained.

### Comparison of performance with other prognostic models

To demonstrate the powerful performance of the prognostic model we have established, we use the R packages “limm’’, “survival”, “survminer” and “timeROC” to compare it with previously published models. The AUC was used to measure the predictive performance of each model.

### GO and KEGG pathway functional enrichment analysis of AGs

To further explore the underlying biological significance of AGs in GC, 39 AGs of the constructed prognostic model were used for functional enrichment analysis. Metascape is an online web tool with comprehensive functions and fast update speed. Therefore, we used this tool to investigate the biological functions and pathways involved in AGs.

### Profiling of infiltrating immune cells

The CIBERSORT algorithm is a linear support vector regression-based machine learning method that accurately identifies the relative abundance of 22 infiltrating immune cells in samples with unknown content and noise (e.g., solid tumors) compared to other deconvolution methods [44]. Therefore, we chose to use the CIBERSORT algorithm to quantify the relative abundance of 22 infiltrating immune cells, including T cells, helper follicular cells and monocytes, in all samples. In addition, to assess reliability of the results, CIBERSORT derives a *P*-value for the deconvolution of each sample using Monte Carlo sampling.

### Building and validating a predictive nomogram

To optimize prognosis prediction, we integrated parameters with independent prognostic significance from multivariate Cox regression analysis to construct nomogram to predict 1-year, 3-year, and 5-year OS survival in GC patients. The calibration curve was utilized to assess the predictive accuracy and discriminatory power of the developed nomogram.

### Statistical analysis

All statistical analyses were performed with R statistical software. Wilcoxon test was used to compare the differences high- and low-risk groups. For all assays, two-sided *P* value of less than 0.05 indicated a statistically significant difference.

## Supplementary Information

**Additional file 1: Figure S1.** The optimal cut-off value of the ARGPs risk-score obtained by the time-dependent ROC curve analysis.

**Additional file 2: Figure S2.** Functional enrichment of 39 unique aging-related genes using the Metascape database. (A) GO enrichment analysis; (B) KEGG enrichment analysis.

## Data Availability

The raw data of this study are derived from the TCGA database (https://portal.gdc.cancer.gov/) and GEO data portal (https://www.ncbi.nlm.nih.gov/geo/), which are publicly available databases.
