# Supplementary figures and images for: A signature of 24 aging‑related gene pairs predict overall survival in gastric cancer
Source: Biomed Eng Online. 2021 Apr 6;20:35. doi: 10.1186/s12938-021-00871-x (PMC8025368; doi:10.1186/s12938-021-00871-x)

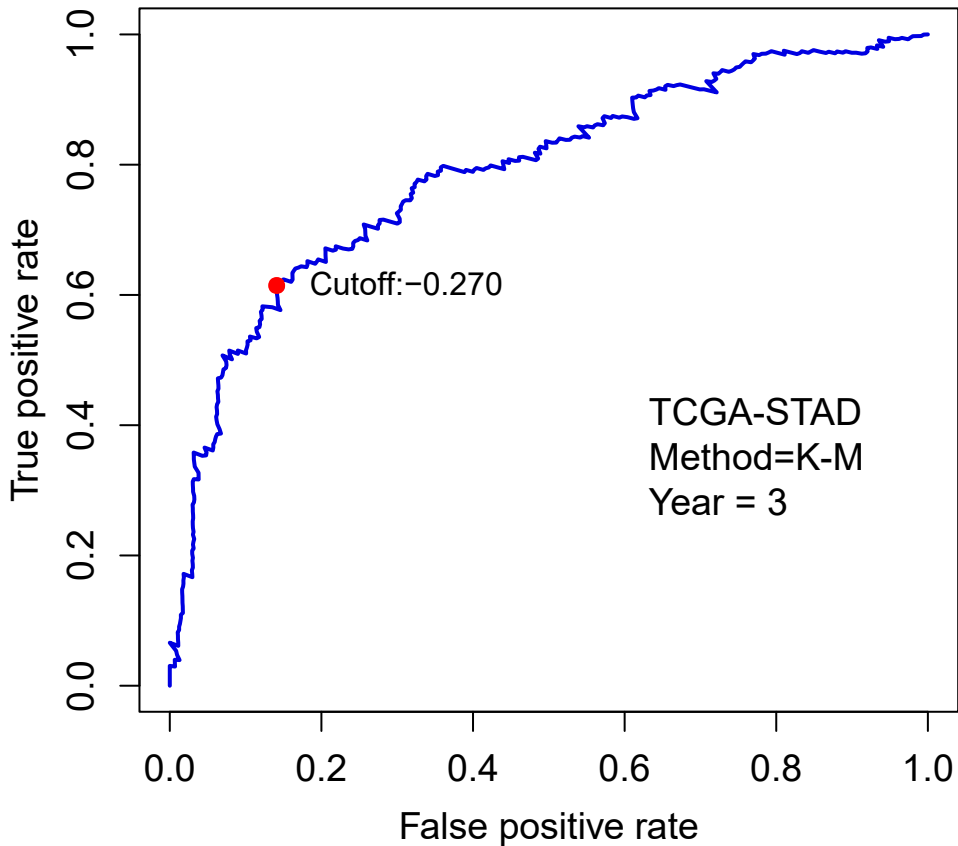

Supplement: Supplementary file 1 — Additional file 1: Figure S1. The optimal cut-off value of the ARGPs risk-score obtained by the time-dependent ROC curve analysis. [file 12938_2021_871_MOESM1_ESM.pdf]
