# Supplementary material for: A signature of 24 aging‑related gene pairs predict overall survival in gastric cancer
Source: Biomed Eng Online. 2021 Apr 6;20:35. doi: 10.1186/s12938-021-00871-x (PMC8025368; doi:10.1186/s12938-021-00871-x)
Supplement: Supplementary file 2 — Additional file 2: Figure S2. Functional enrichment of 39 unique aging-related genes using the Metascape database. (A) GO enrichment analysis; (B) KEGG enrichment analysis. [file 12938_2021_871_MOESM2_ESM.pdf]

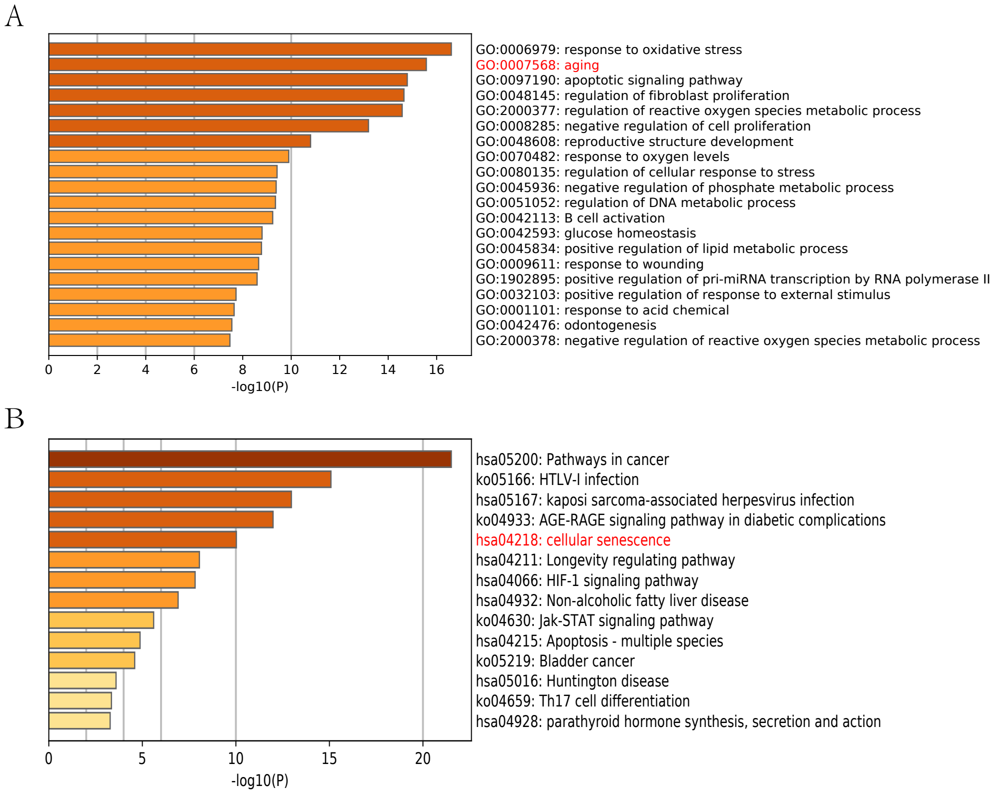
A
GO:0006979: response to oxidative stress
GO:0007568: aging
GO:0097190: apoptotic signaling pathway
GO:0048145: regulation of fibroblast proliferation
GO:2000377: regulation of reactive oxygen species metabolic process
GO:0008285: negative regulation of cell proliferation
GO:0048608: reproductive structure development
GO:0070482: response to oxygen levels
GO:0080135: regulation of cellular response to stress
GO:0045936: negative regulation of phosphate metabolic process
GO:0051052: regulation of DNA metabolic process
GO:0042113: B cell activation
GO:0042593: glucose homeostasis
GO:0045834: positive regulation of lipid metabolic process
GO:0009611: response to wounding
GO:1902895: positive regulation of pri-miRNA transcription by RNA polymerase II
GO:0032103: positive regulation of response to external stimulus
GO:0001101: response to acid chemical
GO:0042476: odontogenesis
GO:2000378: negative regulation of reactive oxygen species metabolic process
0 2 4 6 8 10 12 14 16
-log10(P)
B
hsa05200: Pathways in cancer
ko05166: HTLV-I infection
hsa05167: kaposi sarcoma-associated herpesvirus infection
ko04933: AGE-RAGE signaling pathway in diabetic complications
hsa04218: cellular senescence
hsa04211: Longevity regulating pathway
hsa04066: HIF-1 signaling pathway
hsa04932: Non-alcoholic fatty liver disease
ko04630: Jak-STAT signaling pathway
hsa04215: Apoptosis - multiple species
ko05219: Bladder cancer
hsa05016: Huntington disease
ko04659: Th17 cell differentiation
hsa04928: parathyroid hormone synthesis, secretion and action
0 5 10 15 20
-log10(P)
